# Mixed Parentage Broods Indicate Group Spawning in the Brood Parasitic Cuckoo Catfish

**DOI:** 10.1111/mec.17692

**Published:** 2025-02-17

**Authors:** Holger Zimmermann, Kristina M. Sefc, Radim Blažek, Veronika Bartáková, Anna Bryjová, Cyprian Katongo, Stephan Koblmüller, Martin Reichard

**Affiliations:** ^1^ Institute of Vertebrate Biology Czech Academy of Sciences Brno Czech Republic; ^2^ Department of Biology University of Graz Graz Austria; ^3^ Department of Botany and Zoology, Faculty of Science Masaryk University Brno Czech Republic; ^4^ Department of Biological Sciences University of Zambia Lusaka Zambia; ^5^ Department of Ecology and Vertebrate Zoology University of Lodz Lodz Poland

**Keywords:** African cichlids, Lake Tanganyika, parentage analysis, reproductive parasitism, reproductive success, *Synodontis multipunctatus*

## Abstract

Obligate brood parasites delegate the workload of costly parental care to their hosts. Theory predicts that release from demanding parental care increases the importance of other factors to shape mating patterns. However, behavioural observations and parentage estimates are notoriously difficult to obtain in species with covert reproductive strategies, such as brood parasites, and evidence for their mating strategies are scarce. Molecular genetic methods provide a powerful tool to identify concealed mating patterns. Here, we reconstruct the parentage of cuckoo catfish ( *Synodontis multipunctatus* ) clutches collected in the wild using a combination of newly developed microsatellite markers, mitochondrial markers, and maximum likelihood estimates of pairwise relatedness. Cuckoo catfish parasitise mouthbrooding cichlids in Lake Tanganyika, but a natural spawning of the brood parasite has never been observed. We examined 429 females of confirmed host cichlid species (parasitism prevalence 6%; 24 parasitised clutches with 1–14 embryos) and found that 46% of clutches with three or more offspring (i.e., 6 out of 13) were parented by more than two catfish individuals. We demonstrated variable mating patterns including polyandrous and polygynous mating, and host sharing by separate, genetically monogamous, catfish pairs. This indicates that cuckoo catfish parasitism involves groups of catfish with reduced capability to monopolise mating opportunities. In general, our results demonstrate how reproductive strategy and mating patterns in a species with concealed breeding behaviour can be investigated and provide valuable insights into the mating system of a brood parasitic species other than hitherto studied avian brood parasites.

## 1 Introduction

Mating and brood care behaviour varies widely across animals, including strategies to benefit from the reproductive investment of third‐party conspecific and heterospecific individuals (Royle et al. 2012). Sneaker males, for instance, take advantage of other males' mating success (Taborsky 2008), whilst brood parasites exploit the parental effort of unrelated breeders (Spottiswoode et al. 2012). The latter outsource parental care to other parents of their own or a different species, using strategies termed intra‐ and interspecific brood parasitism, and thereby avoid paying the costs associated with brood care. Interspecific brood parasitism can be obligatory when a species never performs any brood care and is fully dependent on other species for raising their offspring. Amongst vertebrates, this behaviour has repeatedly evolved in birds, with at least seven origins in cuckoos, cowbirds, honeyguides and other avian lineages (Mann 2017). Whilst a lot of research on avian brood parasitism has addressed host–parasite coevolution and associated alloparental care behaviour (Feeney et al. 2014; Soler 2017; Stoddard and Hauber 2017), the behaviour associated with mate choice, mating and egg‐laying of brood parasites is difficult to study due to their stealthy reproductive behaviour. This causes a lack of knowledge of their mating systems (Feeney and Riehl 2019).

Shared parental care often selects for monogamous mating patterns (Wittenberger and Tilson 1980). A release from brood care reduces the benefit of pair bonds in brood parasites, which is predicted to allow for the evolution of more diverse mating patterns, including polygamy and promiscuity (Hauber and Dearborn 2003). Population density, host availability, as well as the need for cooperation and coordination amongst brood parasitic mates to succeed in parasitism (Feeney and Riehl 2019) are important modifiers of their mating system evolution. For instance, limited access to hosts may promote the territoriality of one or both sexes and reduce the probability of polyandrous or polygynous mating (Feeney and Riehl 2019; Hauber and Dearborn 2003). Further, if resources essential for reproduction are scarce or difficult to secure, the need for close cooperation between mates to successfully parasitise hosts may promote monogamy (Hauber and Dearborn 2003).

Tests of theoretical predictions regarding mating patterns of brood parasites are limited to birds, which constrains our general understanding of mating systems of brood parasites (Hauber and Dearborn 2003). Apart from birds, interspecific brood parasitism has been described in insects and a few fish species (Sless et al. 2023). Amongst non‐avian vertebrates, there is only one confirmed obligate brood parasitic species, the cuckoo catfish ( *Synodontis multipunctatus* ). The cuckoo catfish is endemic to Lake Tanganyika, Africa, where it parasitises several mouthbrooding cichlid fishes (Sato 1986). The cuckoo catfish reproductive behaviour provides a useful contrast to avian brood parasites. The major difference between avian and fish brood parasites concerns the mode of egg fertilisation, which happens externally in fish. Hence, both parents must be present during the act of parasitism. In addition, external fertilisation makes fish predisposed to the emergence of alternative reproductive tactics in males (Taborsky 2008).

The mating behaviour of cuckoo catfish is only known from captivity (see video S3 of Blažek et al. 2018 for a video sequence) but has never been observed in nature (Reichard 2019). In aquaria, catfish were observed to intrude on spawning cichlid hosts in groups of 2–6 individuals (when six catfish were present in the aquaria), prey on host eggs and spawn themselves (Zimmermann et al. 2022). Group formation and intrusions often include aggression between the catfish (mainly between the males that join the spawning, pers. observations by authors). For spawning, the cuckoo catfish male clasps the female (termed ‘lock’ as the pair aligns and bends synchronously with the male head positioned in the female abdominal position), followed by egg release and, supposedly, sperm release.

In nature, cichlid hosts reproduce throughout the year and mouthbrooding females of the confirmed host species (Reichard et al. 2023; Takahashi and Koblmüller 2020; Sato 1986) can be observed to breed the entire year (pers. observation by the authors). Most host species are polygynous, with females visiting the territories of several males for spawning, including spawning with different males at multiple locations (Sefc 2011). Courtship and spawning of the cichlid hosts involve distinct behavioural sequences, which are critical for cuckoo catfish intrusions. Male and female cichlids circle above the substrate and the female releases a small batch of eggs, which she immediately collects into her mouth. Fertilisation occurs inside the female buccal cavity when she collects (by mouth) the sperm released by the male. Such distinct spawning bouts, each lasting only a few seconds, are repeated several times during the entire spawning session, which typically takes 1–3 h. In contrast to avian brood parasites, whose host nests are available for parasitism for an extended period of time (egg laying of several days and fixed location of bird nests), cuckoo catfish have to synchronise their spawning with the rather short spawning bouts of their cichlid hosts. Indeed, experiments showed that the timing of the cuckoo catfish spawning is critical for successful parasitism and that it is improved with increasing experience (Zimmermann et al. 2022). Given high fitness cost of catfish parasitism to the host pair (Blažek et al. 2018; Cohen et al. 2019; Reichard et al. 2023; Sato 1986; Zimmermann et al. 2022), the hosts aggressively defend their spawning site from intrusions (Zimmermann et al. 2022) and may reject parasite eggs (Blažek et al. 2018). When disturbed during spawning, the cichlid pair may change spawning location.

The reproductive success of cuckoo catfish depends on egg adoption by the host female. Whilst catfish presumably cannot influence how many parasitic eggs are adopted by the host females, the confusion caused by the catfish intrusion and egg predation makes the cichlid females prone to collect the parasite eggs along with their own (Blažek et al. 2018). It has been hypothesized that larger groups of catfish may be more successful than catfish pairs in overwhelming the host defences and thus facilitating the collection of parasite eggs by the host (Blažek et al. 2021). In laboratory experiments, cuckoo catfish with more experience in interactions with their hosts intrude on host spawnings in groups (i.e., more than two catfish individuals) more often and in larger numbers than naïve catfish (Zimmermann et al. 2022). However, experiments showed no per‐capita difference in parasite reproductive success between treatments with one or three pairs of cuckoo catfish (Blažek et al. 2021). Hence, although the catfish intruded in groups, there was no evidence of a benefit of the potential cooperation.

Reproductive behaviour and mating patterns observed in captivity and natural environment may considerably differ. This is especially pronounced in brood parasitic systems, where host detection and optimal intrusion likely depend on spatial scales such as the population density of the brood parasites and their hosts. Molecular genetic methods provide powerful tools for the study of behaviour, ranging from genomic studies to reveal the evolution of behaviours (Rönkä et al. 2024; Sommer‐Trembo et al. 2024) to the traditional use of simple genetic markers to tackle questions concerning obscured mating behaviours (Flanagan and Jones 2019). To overcome problems arising from the furtive mating behaviour of the cuckoo catfish, we used genetic parentage analysis to understand the mating system of the cuckoo catfish and estimate the number of cuckoo catfish parents contributing to clutches hosted by individual cichlid females. We outline the possible inferences from genetic parentage patterns on the occurrence and composition of catfish spawning groups as follows. (1) If parasite eggs or embryos in the same host were sired by a single male and a single female, catfish either intruded as a pair or a single breeding pair in the group monopolised the reproductive opportunity provided by the spawning cichlid. (2) If parasite eggs or embryos in the same host were sired by a single female and multiple males, cuckoo catfish spawned as a group that either contained only one female or in which one of the females monopolised reproduction. (3) If parasite eggs or embryos in the same host were sired by a single male and multiple females, the spawning group either contained only one male or one of the males was able to monopolise mating. (4) If parasite eggs or embryos in the same host were sired by multiple males and females, cuckoo catfish either spawned as a multi‐male‐multi‐female group and shared reproductive success during the spawning bout, or different pairs intruded sequentially on the cichlid host, possibly when the host female switched between mates. The full‐sib/half‐sib structure of a clutch may distinguish between the two possibilities. Considering that cuckoo catfish reproductive opportunities (i.e., spawning hosts) are a rare resource which are difficult to locate and access, we predicted that the catfish aggregate and spawn in groups in nature, similar to observations in captivity (Blažek et al. 2021; Zimmermann et al. 2022). However, when spawning, competition amongst catfish males for mating as well as competition amongst catfish females for space in the hosts' buccal cavity is high and likely restricts group size. In consequence, we predicted the occurrence of multiple paternity of catfish clutches. We did not necessarily expect multiple maternity of clutches, as females might avoid within‐group competition by asynchronous ovulation (Blažek et al. 2021).

## 2 Methods

Samples were collected from 15 September to 19 October 2019 and from 21 March to 4 April 2022 along the south‐eastern shore of Lake Tanganyika in Zambia (2019: Kalambo Falls Lodge, 8°37′23.3″S, 31°12′00.9″ E; 2022: Mutondwe Island, 8°41′54.6″S, 31°07′04.5″ E, located 12 km apart). To collect catfish clutches, we captured mouthbrooding cichlid females using stop nets (20 m long, 1 m deep and mesh size 6 mm) installed between 1 and 12 m water depth whilst SCUBA diving. Mouthbrooding females can be distinguished by their extended buccal cavity where the brood is located. Females were individually chased by a diver into a stop net, caught using a hand net, and immediately transferred to transparent plastic bags underwater and checked for cuckoo catfish parasitism immediately after the dives. In total, 382 mouthbrooding females of four previously confirmed host species were checked in 2019 ( *Simochromis diagramma* 49 brooding females (9 parasitized), *Shuja horei* 122 (3), *Gnathochromis pfefferi* 144 (3) and *Pseudosimochromis babaulti* 67 (4); Reichard et al. 2023) and 47 mouthbrooding *S. horei* (6 parasitized) in 2022. In 2022, we chose to focus specifically on sampling *S. horei* brooding females for two reasons. First, the species is relatively easy to catch and handle. Second, mouthbrooding females of *S. horei* were more abundant at the diving place than mouthbrooding females of other host species. Since cichlids have a closed swim bladder and need to adjust buoyancy via their blood vessels, females caught below a depth of 6 m were transported to the surface following a decompression period at a depth of approximately 4 m to avoid physical harm to them.

Embryos and eggs were gently flushed out of the host female's buccal cavity using the water jet from a Pasteur pipette. The presence of the cuckoo catfish eggs or embryos was checked visually as both cuckoo catfish eggs and embryos can be clearly distinguished from host eggs and embryos (Cohen et al. 2019; Zimmerman et al. 2023). The cuckoo catfish embryos were euthanized in a bath of overdosed clove oil solution and stored in 96% ethanol until DNA extraction. The host females were released back to the lake after brood extraction.

During field collection in 2019, we additionally collected tissue (fin clips) from 81 adult cuckoo catfish (collected by hand nets and baited traps at Kalambo Falls Lodge and stored in 96% ethanol) to estimate microsatellite marker allele frequencies and test for Hardy–Weinberg equilibrium.

### 2.1 Microsatellite Genotyping

DNA was extracted using the GeneJET Genomic DNA Purification Kit (Thermo Scientific) following the manufacturer's protocol. Parentage analysis was conducted using a new set of commercially developed species‐specific microsatellite markers (AllGenetics & Biology SL; www.allgenetics.eu). In brief, high‐quality genomic DNA from one adult *S. multipunctatus* individual was used to prepare a microsatellite‐enriched genomic library and sequenced using the Illumina NovaSeq platform. A total number of 48 candidate loci were checked for polymorphism in seven *S. multipunctatus* individuals. Excluding markers with fewer than four alleles and markers suspected of harbouring null alleles, a total number of 17 primer pairs were organised into three sets (multiplexes) according to their amplicon size (Table 1). The reverse primers had an oligonucleotide tail at the 5′ end with one of the universal sequences M13 (GGA AAC AGC TAT GAC CAT), CAG (CAG TCG GGC GTC ATC) or T3 (AAT TAA CCC TCA CTA AAG GG). Oligonucleotides with sequences corresponding to the 5′‐tails of the reverse primers were used to label the amplicons with the fluorescent dye (VIC, FAM, NED) during PCR.

**TABLE 1:** Characteristics of microsatellite loci used for parentage analysis.

Loci	Primer sequence 5′–3′	Repeat motif	*n*	*N*_A_	Dye	Size range (bp)	*H*_O_	*H*_E_	*P*_HWE_	Multi
Smu012	F: TTGTTCGGCCTCTTGTCTCT	(AC)_n_	81	12	F	202–234	0.73	0.8	0.278	1
	R: CCGAGCAGGCAACTAATCTC									
Smu071	F: AAATGGGACTGATACTGCCG	(AC)_n_	81	15	N	132–164	0.82	0.84	0.721	1
	R: TTTCATTGCTTGGGACCTTC									
Smu085	F: ATTTCCTCTCACCTCGGGTT	(AATG)_n_	81	5	F	319–335	0.6	0.68	0.052	1
	R: CACGTATTCCATTCGCCTTT									
Smu228	F: TCTCCACCACTCACTGCAAG	(AC)_n_	81	20	V	95–139	0.96	0.93	0.766	1
	R: GCATCAGCTCATGTTTGCAT									
Smu375	F: TTCGGCTTTATCCTGGTCTG	(AC)_n_	81	8	N	306–330	0.73	0.75	0.375	1
	R: TCAATCAACAGTGGCATGGT									
Smu456	F: GCAAACAAGCACTTTAGCCC	(AG)_n_	81	21	N	186–224	0.9	0.91	0.426	1
	R: CTGCTCCCTGCTCTTACTGG									
Smu006	F: GAAACATAAGCAGGCGCTTC	(AG)_n_	81	8	N	228–252	0.72	0.76	0.365	2
	R: ACATTGACACGTCCACTCCA									
Smu136	F: GTTTGGCCAACCAACATACC	(AC)_n_	81	19	N	83–129	0.85	0.83	0.573	2
	R: CGATGTGCCCTATTTCAGGT									
Smu198	F: GTCAGGCTGGTGATGGATCT	(AC)_n_	81	8	F	208–228	0.63	0.64	0.953	2
	R: AGCTTTATGTGCCGTTGCTT									
Smu280	F: GTGGTGAACCCTAGTTGGGA	(AGG)_n_	81	5	V	133–151	0.32	0.33	0.834	2
	R: TACAAACAAACCGGAAAGCC									
Smu408	F: CCCTTCCAAAGCCTTAAACC	(AG)_n_	81	18	F	296–334	0.93	0.91	0.470	2
	R: GCACGCAATCATTTCTCAAA									
Smu185	F: AACAAATGGGCCAATCAGAG	(AC)_n_	81	7	F	216–228	0.55	0.51	0.621	3
	R: CTACCGATGCATTCGAGGTT									
Smu308	F: GGCAGCACAGACCGATTTAT	(AC)_n_	81	4	F	199–205	0.62	0.6	0.559	3
	R: TGGATTGTGGTAGGTGCTGA									
Smu318	F: TGACTGCAGTGCCACTCTTC	(AC)_n_	81	16	V	204–236	0.89	0.86	0.181	3
	R: TTTGCTCACTGCCACTTCAC									
Smu328	F: ACCGTGACATCATCAGGGAT	(ATC)_n_	81	4	F	262–278	0.48	0.49	0.816	3
	R: CTCAGAGGAACCTGCACTCC									
Smu357	F: AAACTGGATCCCAGTGCAAC	(AG)_n_	81	22	N	238–274	0.88	0.89	0.062	3
	R: ATTTGACCAATCTGGCAACC									
Smu481	F: CCACGCTAGGCATTTAGAGC	(AC)_n_	81	7	V	118–132	0.75	0.77	0.589	3
	R: GAACCTGTTCAGCAGCCATT									

*Note:* Dye: fluorescent dye used to detect fragments; *N* = NED, V = VIC, F = FAM.

Abbreviations: *H*_E_, expected heterozygosity; *H*_O_, observed heterozygosity; HWE, Hardy–Weinberg equilibrium; Multi, multiplex identification number; *N*_A_, number of alleles.

PCR was performed in 10 μL volume, containing 1 μL of DNA, 5 μL of Multiplex PCR Master Mix (Qiagen) and Primer Mix 1× (0.1–0.75 μM forward primers and 0.1–1.1 μM labelled tails, and 0.01–0.075 μM reverse primers). The thermocycler protocol consisted of an initial denaturation step at 95°C for 15 min, followed by 30 PCR cycles (95°C for 30 s, 57°C for 90 s and 72°C for 30 s) and 8 PCR cycles with reduced annealing temperature (95°C for 30 s, 53°C for 90 s and 72°C for 30 s), and a final extension at 68°C for 10 min. Electrophoresis of PCR products was carried out in an ABI 3130 Genetic Analyser (Applied Biosystems, Foster City, California, USA) and alleles were sized against the internal size standard (GeneScan LIZ‐500) using the Peak Scanner Software 2.0 (Applied Biosystems).

Genotypes were produced for 93 cuckoo catfish embryos (2019: 60 embryos from 19 clutches, 2022: 33 embryos from 5 clutches). We used the software ‘identity4’ (Wagner and Sefc 1999) to estimate population allele frequencies from the 81 samples of adult cuckoo catfish. Deviations from Hardy–Weinberg equilibrium were tested in Arlequin vs. 3.5.2.2 (Excoffier and Lischer 2010).

### 2.2 Sequencing of the Mitochondrial Control Region

Primers for part of the mitochondrial control region (i.e., D‐loop region used for determination of different cuckoo catfish females) were designed based on whole mitogenome sequences of the congeneric species *Synodontis schoutedeni* and *Synodontis clarias* (GenBank Accession Numbers: AP012023, NC_015808, OL450422 and NC_063752) using Geneious 9.0.5 software (https://www.geneious.com). The newly designed primers are synoDL2F (forward, 5′‐CCTAACTCCCAAAGCTAGGATTC‐3′) and synoDL2R (reverse, 5′‐GATTGAGGGCATTCTCACAGG‐3′). PCR was performed in 10 μL volume, containing 1.5 μL DNA extract, 10 μM forward primer, 10 μM reverse primer, 5 μL Multiplex PCR Master Mix (Qiagen) and 3 μL ddH_2_O. The thermocycler conditions were as follows: initial denaturation at 95°C for 15 min, followed by 35 cycles of 94°C for 30 s, 58°C for 30 s, 72°C for 1 min and final extension at 72°C for 10 min. The PCR products were purified with FastAP Thermosensitive Alkaline Phosphatase (ThermoScientific) and Exonuclease I (ThermoScientific) and sequenced by Eurofins Genomics Germany GmbH (www.eurofinsgenomics.eu). Sequences were obtained from 51 cuckoo catfish embryos, including all clutches with three or more embryos in 2019 and one clutch in which monogamous mating was not supported in 2022 (i.e., 8 clutches in 2019 and 22.SH02 in 2022, see Table 2) and were deposited in GenBank (GenBank Accession Numbers OR208216, OR208219–OR208222, OR208224–OR208227, and PQ112701–PQ112743). Sequences were aligned in MEGA11 (Tamura et al. 2021) and assigned to haplotypes using the software DnaSP v6 (Rozas et al. 2017). To visualise the relationships amongst the different haplotypes, a neighbour joining tree (based on the Maximum Composite Likelihood method) was inferred in MEGA11.

**TABLE 2:** List of cuckoo catfish clutches examined for multiple parentage. The table reports the year of collection (sample year), the host species, the number of catfish embryos and their identification codes (consisting of sampling year, host species initials and letters denoting individual embryos with the clutch).

Sample year	Host species	Number of catfish embryos	Catfish embryo ID
2019	*Gnathochromis pfefferi*	4	19.GP01.A–19.GP01.D
2019	*Simochromis diagramma*	3	19.SD01.A–19.SD01.C
2019	*Gnathochromis pfefferi*	14	19.GP02.A–19.GP02.N
2019	*Simochromis diagramma*	5	19.SD02.A–19.SD02.E
2019	*Shuja horei*	3	19.SH01.A–19.SH01.C
2019	*Simochromis diagramma*	3	19.SD03.A–19.SD03.C
2019	*Simochromis diagramma*	5	19.SD04.A–19.SD04.E
2019	*Simochromis diagramma*	8	19.SD05.A–19.SD05.H
2022	*Shuja horei*	8	22.SH01.A–22.SH01.H
2022	*Shuja horei*	7	22.SH02.A–22.SH02.G
2022	*Shuja horei*	7	22.SH03.A–22.SH03.G
2022	*Shuja horei*	5	22.SH04.A–22.SH04.E
2022	*Shuja horei*	6	22.SH05.A–22.SH05.F

### 2.3 Assignment of Parentage

To avoid false inferences of group spawning, our parentage analysis aimed for the most parsimonious reconstruction of catfish parentage amongst the sampled clutches. We therefore searched for the minimum number of dams and sires compatible with the embryo genotypes in each clutch in COLONY 2.0 (Jones and Wang 2010), where ‘clutch’ refers to the catfish embryos co‐hosted by a particular cichlid female. Given that parental genotypes were not known in our study, multiple parentages could only be inferred in clutches with three or more embryos because any two embryo genotypes can always be explained by a single‐parent pair. This yielded a sample of 13 clutches (of a total of 24 clutches; 19 clutches from 2019 and 5 from 2022) in which multiple parentage could be tested (Table 2).

To reconstruct the most parsimonious parent numbers for these 13 clutches, we first used the maximum likelihood analysis of COLONY 2.0 to assign embryos into full‐sib and half‐sib groups. To minimise presumptions of allele sharing between inferred parents, which can lead to an overestimation of dam and sire number, we set the frequencies of all observed alleles to 0.001 (Sefc and Koblmüller 2009). Almost identical results were obtained when using the actual allele frequencies estimated in the population sample (result not shown). Sibship reconstruction was conducted in two pooled samples containing the catfish embryos sampled in 2019 (59 embryos from 19 clutches) and in 2022 (33 embryos from 5 clutches), respectively. The parentage of embryos in the 13 clutches containing three or more embryos was assigned based on the reconstructed sibship structure. For instance, if a clutch consisted of two full‐sib groups and the two full‐sib groups were each other's half sibs, then this clutch was assumed to descend from three parents, one of which was shared by all embryos. The microsatellite data do not indicate which parent is the sire and which is the dam. We therefore used partial mitochondrial control region sequences (see above) to distinguish between multiple paternity and multiple maternity in clutches with more than two parents. When half sibs had different mitochondrial haplotypes, they were assumed to have different mothers and share a common father. This method is limited by the possibility that two females share the same haplotype, impeding clear assignment. We therefore estimated the haplotype diversity within our sample to validate our approach. A sample of 19 catfish embryos collected and sequenced in a previous study (Reichard et al. 2023), which consisted of one randomly selected embryo per co‐hosted clutch, contained 13 different haplotypes (same mitochondrial locus and same catfish population as used here, File S1). Haplotype diversity amounted to 89%, suggesting a high likelihood of discriminating different females based on their mitochondrial sequences. The next step consisted of manual control of whether genotypes detected in clutches, for which three or more parents were reconstructed by COLONY, could also be explained by a lower number of parents. This could be due to the over‐splitting of embryos into different sib groups (Sefc and Koblmüller 2009) but also due to genotyping errors. We therefore also re‐checked the electropherograms in cases where an extra parent was based on three or fewer allelic mismatches.

Finally, we examined whether the inferred maternal and paternal kinship amongst embryos was compatible with the likelihoods of different kinship types (full sib, half sib, and unrelated) calculated in the software ‘ML‐Relate’ (Kalinowski et al. 2006), and performed likelihood ratio tests to distinguish between alternative kinship types. Briefly, ML‐Relate uses the scored genotypes of a population sample (i.e., 81 samples of adult cuckoo catfish from 2019) to calculate allele frequencies. Then, it estimates the maximum likelihood for given types of kinship (unrelated, half sib, full sib and parent–offspring) for all dyads (i.e., all cuckoo catfish embryos collected in 2019) and performs likelihood ratio tests to distinguish between alternative kinship types (10,000 simulations for each dyad). We did not consider parent–offspring relationships as meaningful results as all samples were embryos and included these dyads as full sibs to the results.

## 3 Results

### 3.1 Microsatellite and Mitochondrial Sequence Markers

Our set of 17 microsatellite markers provided satisfactory power to resolve parentage in our dataset (overall exclusion probability > 0.99999). The observed and expected heterozygosity of individual markers ranged from 0.32 to 0.96 and from 0.33 to 0.93, respectively. Mean (±S.E.) observed and expected heterozygosity were 0.73 (±0.043) and 0.74 (±0.042) (Table 1). Number of alleles ranged from 4 to 22 alleles per locus, with a mean (±S.E.) of 11.7 (±1.6). None of the loci deviated from Hardy–Weinberg equilibrium (Table 1).

The alignment of the mitochondrial control region sequences comprised 596 bp after the removal of ambiguously aligned regions and positions with gaps or missing data. The examined subset of clutches (*n* = 9 clutches, 51 embryos) contained a total of 7 different haplotypes (File S2, Figure S1). Five of the clutches contained more than one haplotype, indicative of multiple maternity.

### 3.2 Parentage Analysis

**FIGURE 1:**
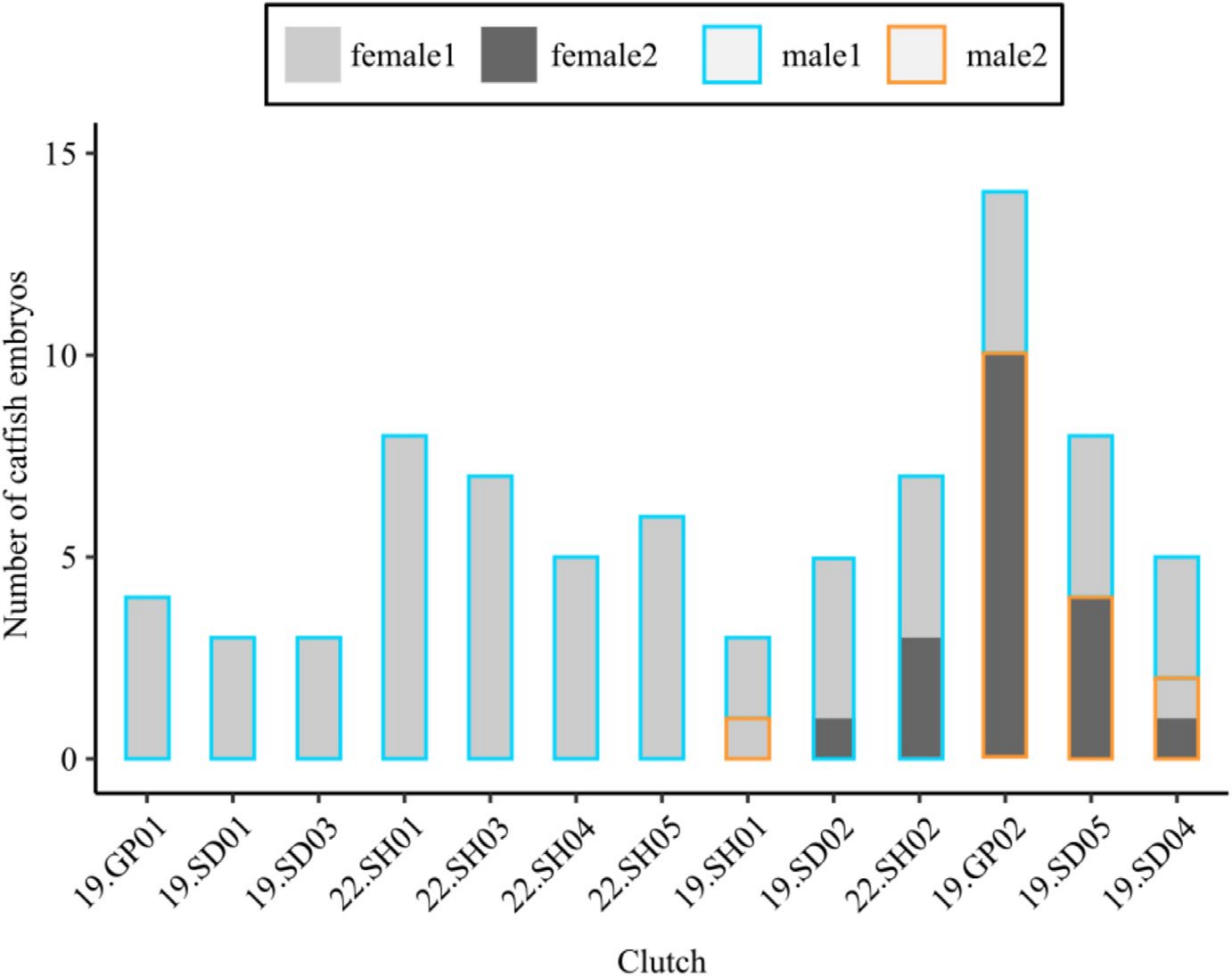
Parentage of *Synodontis multipunctatus* clutches. For each clutch, the bars illustrate the assignment of catfish embryos to dams (differentiated by bar colours) and sires (differentiated by contour line colours) according to the most parsimonious parent number reconstruction. Only analysed clutches (i.e., clutches > 2 embryos) are shown.

**FIGURE 2:**
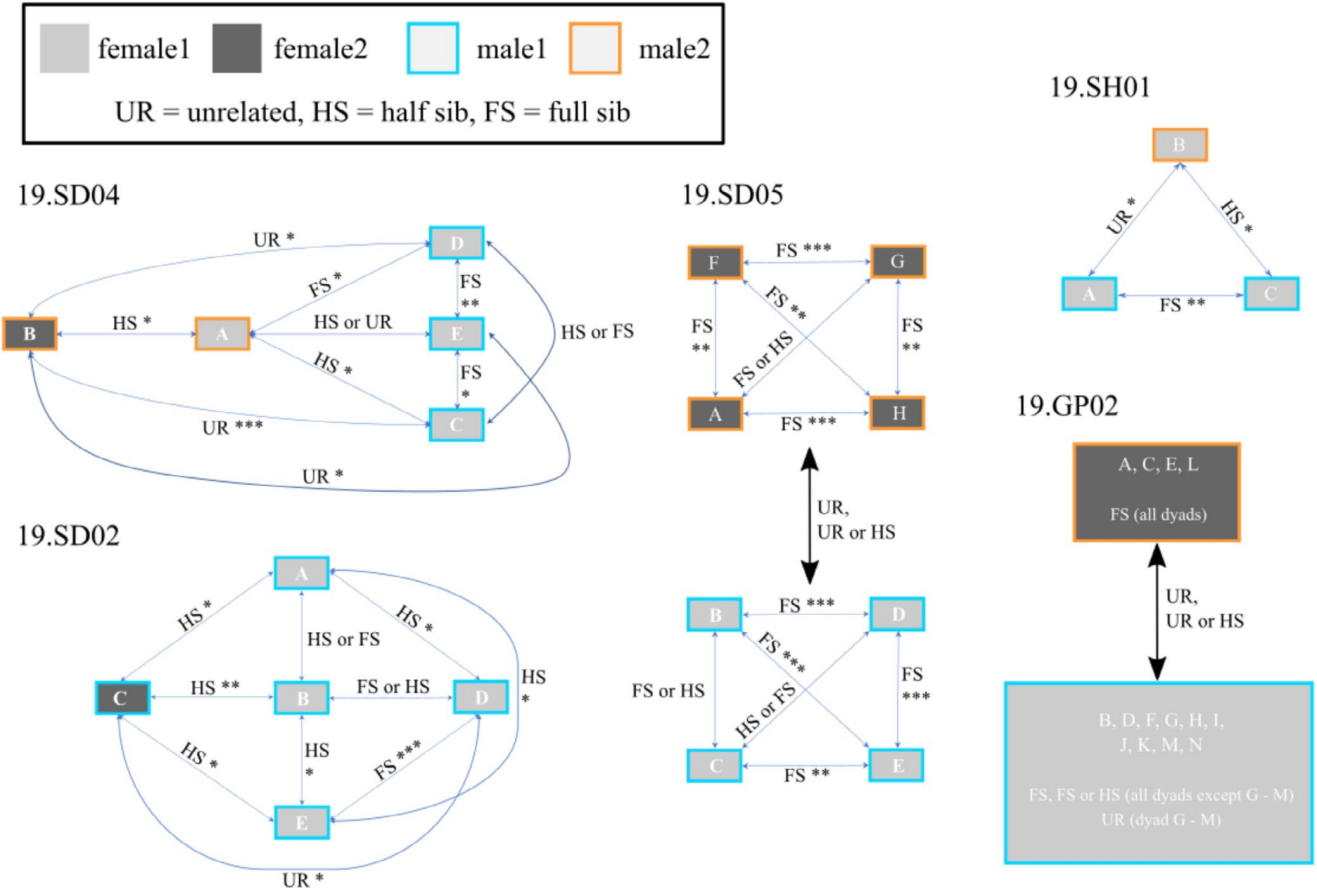
Dyadic kinship amongst *S. multipunctatus* embryos. For each of the clutches (except 19.GP02) included in the ML‐Relate analysis, the plots depict catfish embryos (labelled A, B, C, etc.) and their most likely relationship. The relationship was inferred by likelihood ratio tests comparing alternative putative relationships. When the statistical test identified one significantly most likely relationship, the line connecting the embryos is labelled by that relationship code and the significance level (*p < 0.05, **p < 0.01, ***p < 0.001) at which the second most likely relationship type was rejected. When the test failed to differentiate between two alternative types of relationship (*p* > 0.05), both are indicated (e.g., “HS or FS”). The colours of the embryo boxes and the contour lines reflect the parentage reconstruction shown in Figure 1. The illustration of clutch 19.GP02 is simplified due to the high number of offspring in this clutch and depicts the two full‐sib groups reconstructed by COLONY. Likelihood ratio tests were consistent with relationship type UR for all dyads across the two full‐sib groups. Within full‐sib groups, likelihood ratio tests were consistent with the full‐sib relationships of all but one dyad (embryos G–M: significantly most likely relationship = UR).

Thirteen broods (8 in 2019, 5 in 2022, Table 2) contained more than two cuckoo catfish embryos (median = 5 embryos, range = 3–14 embryos). The COLONY analysis inferred multiple parentage in six of these clutches (46%; Figures 1 and 2), with 3 or 4 parents per clutch. Across the whole dataset (19 clutches from 2019 and 5 clutches from 2022), COLONY assigned some embryos (in 2019 and especially those in small clutches with 1 or 2 individuals) as half sibs to embryos from other clutches (Table S1), but reconstructed full sibs were never distributed across different host individuals.

For one of the clutches used for parentage reconstruction (i.e., clutches containing three or more embryos), 19.SH01, the COLONY analysis suggested that the three embryos of this clutch might be half sibs produced by two dams and two sires (Table S1). However, all embryos shared the same mitochondrial haplotype (Figure 2, File S2) and the microsatellite genotypes of the embryos could be explained by three parents. We therefore parsimoniously assumed this clutch to descend from one mother and two fathers (Figure 1), although the alternative reconstruction involving two mothers sharing the same haplotype cannot be excluded. For clutch 19.SD04, the COLONY sibship reconstruction required five parents (Table S1), whereas a manual re‐arrangement of parentage assignments arrived at minimum estimates of two dams (distinguished by microsatellite alleles and mitochondrial haplotypes) and two sires (Figures 1 and 2). One of the two dams in this clutch was inferred to have spawned with both males, and one of the two males had spawned with both females (Figure 1). The other four multiply parented clutches each contained two mitochondrial haplotypes (Figures 1 and 2) and indicated either mating of two separate monogamous pairs (two full‐sib groups in clutches 19.GP02 and 19.SD05) or two dams sharing one sire (i.e., paternal half sibs, clutches 19.SD02 and 22.SH02) (Figure 1).

Kinship hypothesis tests in ML‐Relate (comparing the maximum likelihood of alternative pairwise relationships) were largely congruent with the above parentage assignments (Figure 2, Table S2). In some cases, the most likely kinship of one dyad conflicted with the most likely kinship of another dyad. These conflicts occurred in clutches 19.SH01 and 19.SD04, in each of which one of the embryos (B in 19.SH01 and A in 19.SD04) was inferred to have different types of kinship to individual members of a co‐hosted full sib group. These were the clutches in which the most parsimonious parent number reconstruction differed from the COLONY parentage reconstruction. The ML‐Relate analysis did not suggest stronger evidence in favour of any of the two solutions. For clutch 19.SD02 reconstruction of parentage was more complicated. Whilst the COLONY results supported three parental individuals for the embryos, ML‐Relate results remained ambiguous with weakly supported kinship dyads for the larger full‐sib group (embryos 19.SD02. A, B, D and E), indicating two maternal females which shared the D‐loop haplotype. Nevertheless, since two of the embryo dyads remained inconclusively resolved, we decided to assume the result with the minimum number of parents as the most parsimonious (Figure 2).

## 4 Discussion

We reconstructed parentage in cuckoo catfish clutches collected in their natural environment to investigate mating patterns of the brood parasitic catfish and the possibility of cooperative or competitive group intrusions to cichlid host spawnings. Group intrusions on cichlid hosts have previously been recorded in captivity (Blažek et al. 2021; Zimmermann et al. 2022), but the cuckoo catfish reproductive behaviour has never been observed in nature. Group intrusions to host nests are a common strategy of at least one cowbird brood parasite, probably to increase host nest accessibility (Robinson 1988). Understanding behavioural, spatial and temporal patterns of mating is often impeded by covert mating behaviour. Molecular genetic methods provide excellent tools to overcome the lack of robust behavioural data provided that a representative sample of offspring can be obtained. Our analysis confirms group intrusions in the cuckoo catfish by demonstrating polyandrous, polygynous and polygynandrous mating based on the sibship reconstructions within catfish clutches (Table 3) and demonstrates great variation in cuckoo catfish mating patterns.

**TABLE 3:** Summary of parentage reconstruction. The first two columns report the reconstructed mating patterns and the most parsimonious number of parents per clutch. The third column refers to the scenarios outlined in the introduction and assigns the parentage reconstructions to the corresponding scenarios. The fourth column reports the number and identity of catfish clutches representing the inferred parentage and mating patterns with the number of embryos for each clutch reported in parentheses.

Mating pattern	Number of parents	Number of mitochondrial haplotypes	Inference	Catfish clutches
Monogamous mating of one pair, clutch consists of 1 FS group	1 female, 1 male	1	Pair spawning, or one reproducing pair in the spawning group	7 clutches: 19.GP01 (4), 19.SD01 (3), 19.SD03 (3), 22.SH01 (8), 22.SH03 (7), 22.SH04 (5), 22.SH05 (6)
Polyandrous mating	1 female, 2 males	1	Spawning group	1 clutch: 19.SH01 (3)
Monogamous mating of two pairs, clutch consists of 2 FS groups	2 females, 2 males	2	Spawning group, or consecutive pair spawning	2 clutches: 19.GP02 (14), 19.SD05 (8)
Polygynous mating	1 male, 2 females	2	Spawning group	2 clutches: 19.SD02 (5), 22.SH02 (7)
Polygynous and polyandrous mating	2 females, 2 males	2	Spawning group	1 clutch: 19.SD04 (5)

Polyandrous mating may arise from the existence of male alternative reproductive tactics (Taborsky 2008). The cuckoo catfish mating behaviour includes close contact between male and female and, in captivity, reproductively active males sometimes clasp each other (HZ, pers. obs.). Hence, mating trios are a possible configuration for the cuckoo catfish spawning behaviour. Alternatively, a catfish female may use two separate cichlid egg‐laying bouts within a single cichlid spawning event to mate with two different males. Polygynous mating, in contrast, requires that one male mates with two different females consecutively, which is feasible given the cichlid host's spawning procedure consisting of several separate egg‐laying sessions. Finally, the presence of two unrelated full‐sib groups within the same host (Table 3) implies that they were produced by catfish groups consisting of at least two males and two females, or that different spawning pairs intruded consecutively on the same cichlid host. The complex parentage pattern, including multiple maternity and paternity, of brood 19‐SD04 suggests that at least in one case, simultaneous spawning of two males and two females was likely. The remaining seven clutches (with three or more parasite offspring) descended from a single‐parent pair. Overall, it is clear that at least some clutches included group intrusions and hence, they occur under natural conditions.

Congregating in groups brings several potential benefits for the group members. First, groups may be more successful in detecting spawning cichlids, similar to avian brood parasites (Hauber and Dearborn 2003) and analogous to the function of bird foraging aggregations (Lack 1968; Ward and Zahavi 1973). Groups may also be more successful than pairs in overcoming the cichlids' defences against intrusion. As a consequence, all catfish group members would benefit from improved opportunities to prey on cichlid eggs (Reichard 2019; Zimmermann et al. 2022), and the spawners additionally benefit from easier reproductive opportunities. Additionally, as the cuckoo catfish must learn to precisely modulate their own gamete release to the most appropriate time window of host spawning (Zimmermann et al. 2022), younger individuals should benefit from the association with more experienced breeders to develop their competence to parasitise (Valone 2007).

Cooperation has evolved repeatedly in contexts like hunting for prey (Bshary et al. 2006; Packer and Ruttan 1988), defence against predators (Farabaugh et al. 1992; Krakauer 1995), courtship (Krakauer 2005; Taborsky 1994) and brood care (Burt et al. 2007; Wong and Balshine 2011). The potential hosts of avian brood parasites recognise the risk of parasitism and mob potential brood parasites in the vicinity of their nests (Medina and Langmore 2016; Welbergen and Davies 2009). Arguably as a response to host defence, the brood parasitic giant cowbirds ( *Molothrus oryzivorus* ) sometimes intrude on host colonies in small groups, with females coordinating their attempts to lay their eggs and males distracting the host individuals (Robinson 1988; Webster 1994). Further, in the host‐specific screaming cowbird ( *Molothrus rufoaxillaris* ), males accompany females when visiting host territories (Scardamaglia and Reboreda 2014), possibly to assist in the location of prospective host nests (De Mársico et al. 2019). Cooperation between males and females has also been observed in some other brood parasitic bird species (reviewed in Feeney and Riehl (2019)), but the cryptic behaviour and concealed egg laying of these species limit systematic examinations and reports often remain anecdotal (Feeney and Riehl 2019).

It is likely that group intrusions by the cuckoo catfish are much more common than the three polygamous clutches indicate. This is because it is conceivable that only a few eggs spawned by catfish are successfully adopted and incubated by the host. The hosts may by chance pick up eggs from only one of several spawning catfish pairs. Of the successful subset of parasite eggs, mortality and egg rejection during the mouthbrooding period (Blažek et al. 2018) may eliminate the offspring of other group members. More than half of the catfish clutches collected in the wild contained only one or two embryos (Reichard et al. 2023), suggesting that a considerable proportion of catfish eggs and embryos may not be collected (or accepted) by the hosts or fails to survive. Another source of skew in reproductive success may result from competition over mating opportunities and variation in the experience amongst intruding catfish. Dominant males may succeed in monopolising access to females, and dominant or experienced females may be particularly successful in placing eggs to maximise the chances for their adoption by the cichlid host. However, our results indicate the occurrence of monogamous, polygynous and polyandrous mating by cuckoo catfish, suggesting variable mating behaviour and low ability to monopolise mating opportunities.

We hypothesised that competition for mates (in males) and restricted space in the hosts´ buccal cavity (in females) likely restricts cuckoo catfish group size. The observation that none of the clutches involved more than two sires and two dams agrees with this assumption, although the small size of many examined catfish clutches (median clutch size of 5 eggs) and our conservative approach to parent number estimation may bias the estimates downwards. Competition amongst group members restricts group size if fitness gains expected from group membership decrease with increasing numbers of competing group members (Clutton‐Brock 2016, pages 12, 63, 289). Indeed, whilst group intrusions bear clear benefits for cuckoo catfish, per‐capita reproductive output was independent of catfish group size in an experimental situation. Moreover, the numbers of parasite eggs retrieved from individual hosts did not differ between low‐ and high‐parasite density treatments, such that there was no evidence for multiple parasitism of the same host individual (Blažek et al. 2021). This suggests that easier access to potential hosts, which may be particularly relevant under field conditions, may be countered by intra‐group competition for mating partners and for space in the host's buccal cavity. In addition, females might avoid intra‐group competition by asynchronous ovulation of their eggs (Blažek et al. 2021), although we found several clutches produced by two females.

When brood parasites evolve from care‐giving ancestors, the release from parental care duties is expected to favour polygamy (Hauber and Dearborn 2003). Empirical studies demonstrated the effects of additional factors such as the home range size of the avian brood parasites, parasite population density, and the spatial distribution of their hosts (reviewed in Feeney and Riehl 2019). For example, mating systems in greater spotted cuckoos vary amongst populations from predominantly monogamous to highly polygamous according to parasite population density (Bolopo et al. 2017). In Horsfield's bronze‐cuckoos, pairs occupy large non‐overlapping breeding ranges, including several host territories and are strictly monogamous (Langmore et al. 2007). In contrast, the hosts of the related little bronze‐cuckoos occur more concentrated in suitable habitat patches, which leads to a higher density of brood parasites and facilitates polygamous mating (Noh et al. 2024). The brood parasitic cuckoo catfish evolved from non‐caring ancestors (Reichard 2019), and the mating patterns of non‐parasitic (egg scattering) *Synodontis* catfish are not known. Hence, the ancestral state of cuckoo catfish mating behaviour remains unresolved. Given that the requirements for successful parasitism in birds and the cuckoo catfish are similar (regarding the localisation of suitable hosts, as well as the effects of parasite and host densities), it seems feasible that the same environmental factors play a role in their mating patterns. Contrary to avian brood parasitic species, both parasite parents must be present during the parasitic event because the fertilisation of eggs happens externally at the timepoint of parasitism. This may facilitate monogamous matings and the occurrence of extra‐pair paternity caused by sneaker males in cuckoo catfish. Without observations of the analysed cuckoo catfish spawnings, our results support high plasticity in mating patterns similar to that reported in the greater spotted cuckoos (Bolopo et al. 2017).

It has been proposed that the brood parasitism of the cuckoo catfish evolved from catfish predation on cichlid eggs (Mouginot et al. 2024). Egg predation during cichlid spawning elicits the hasty collection of eggs by the spawning cichlid female, a situation that may be exploited by the catfish to present their eggs to the mouthbrooding cichlids. Group intrusions may have already been favoured at this early stage of catfish‐cichlid interactions, initially perhaps simply to overwhelm anti‐predatory defence and increase the egg predation success. This may have allowed some group members to take advantage of the confusion for their own reproduction, perhaps facilitated by the presence of host reproductive hormonal stimulation (Mouginot et al. 2024).

In the layout of this study, we outlined possible parentage patterns of the cuckoo catfish clutches (i.e., monogamous vs. polyandrous and/or polygynous mating) and associated them with different inferences for catfish mating behaviour. Surprisingly, the detected parentage patterns exceeded our expectations concerning their variability by covering the whole range of possible mating scenarios. Whilst parentage patterns in cuckoo catfish clutches do not only reflect mating behaviour but also differential egg adoption by hosts and mortality of embryos within the host buccal cavity, our data provide considerable insight into the covert reproductive behaviour of these brood parasites. In particular, the data provide clear support for the occurrence of group intrusions by brood parasitic catfish but also suggest that either group size or the proportion of successfully reproducing group members is limited. In the future, estimates of relatedness within intruding groups can provide information on potential for kin selection. Whilst we uncovered mating patterns in a species with concealed breeding behaviour, it remains to be revealed whether group intrusions are a typical or occasional situation in the natural reproductive behaviour of the cuckoo catfish.

## Author Contributions

M.R., K.M.S., S.K. and H.Z. conceptualised and designed the study. M.R., S.K., H.Z., V.B. and R.B. conducted field work. C.K., S.K. and M.R. were involved in project administration. V.B. and A.B. conducted lab work. H.Z. and A.B. genotyped samples. H.Z. and K.M.S. analysed data. H.Z., M.R. and K.M.S. wrote the manuscript with significant contributions from all co‐authors.

## Conflicts of Interest

The authors declare no conflicts of interest.

## Benefit Sharing Statement

Benefits Generated: This research is part of a long‐term collaboration between European and Zambian partners within the framework of a Memorandum of Understanding (MoU). It includes a co‐author from the country where research was conducted, and contributing institutions were acknowledged in the Acknowledgement section of this paper. Our research group is committed to international scientific partnerships and capacity building. All data have been shared with the broader scientific community via appropriate biological databases.

## Supporting information


Figure S1

Table S1

Table S2

File S1

File S2

## Data Availability

Unique sequences of the partial control region were uploaded to NCBI GenBank under the accession numbers PQ112701–PQ112743. Individual genotypes and unique haplotype sequences are accessible on FigShare repository (https://doi.org/10.6084/m9.figshare.28296464).
