# Supplementary material for: Mixed Parentage Broods Indicate Group Spawning in the Brood Parasitic Cuckoo Catfish
Source: Mol Ecol. 2025 Feb 17;34(6):e17692. doi: 10.1111/mec.17692 (PMC11874674; doi:10.1111/mec.17692)
Supplement: Supplementary file 1 — Figure S1 [file MEC-34-e17692-s001.docx]

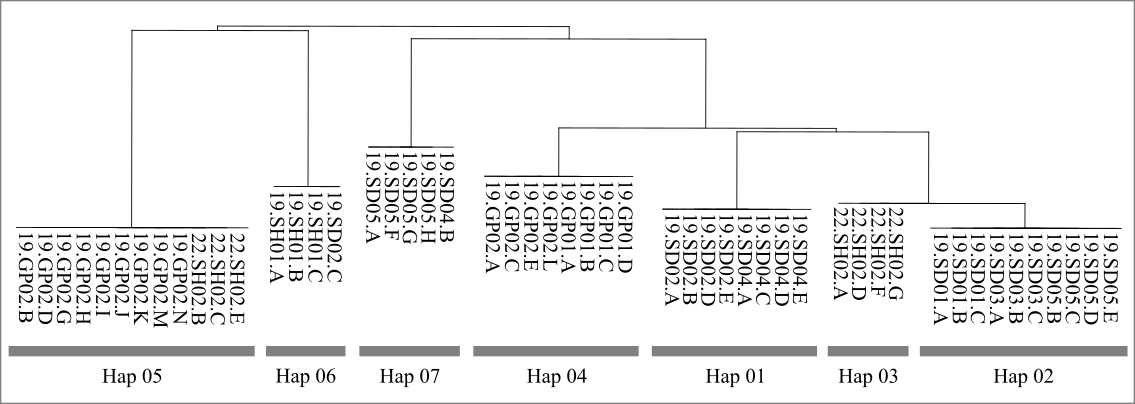


**Supplementary Figure 1:** Neighbour joining haplotype tree of cuckoo catfish clutches. The haplotype tree was calculated in MEGA11 (Tamura, Stecher, & Kumar, 2021) excluding gaps and missing data.
