# Supplementary material for: Mixed Parentage Broods Indicate Group Spawning in the Brood Parasitic Cuckoo Catfish
Source: Mol Ecol. 2025 Feb 17;34(6):e17692. doi: 10.1111/mec.17692 (PMC11874674; doi:10.1111/mec.17692)
Supplement: Supplementary file 2 — Table S1 [file MEC-34-e17692-s003.pdf]

Supplementary Table 1: Results of the two Colony analyses for cuckoo catfish clutches collected in 2019 and 2022 respectively. For the Colony runs, all observed allele frequencies were set to 0.001 to avoid overestimates of parent numbers and polygamy was allowed for both sexes.

collection date	*embryo ID*	*Parent 1*	*Parent 2*
2019	19.GP01.A	*1	#1
2019	19.GP01.B	*1	#1
2019	19.GP01.C	*1	#1
2019	19.GP01.D	*1	#1
2019	19.SD01.A	*2	#2
2019	19.SD01.B	*2	#2
2019	19.SD01.C	*2	#2
2019	19.SD06.A	*3	#3
2019	19.SD06.B	*3	#3
2019	19.GP13.A	*4	#4
2019	19.CH28.A	*5	#5
2019	19.SD07.A	*3	#6
2019	19.SD07.B	*3	#6
2019	19.CH45.A	*4	#7
2019	19.GP02.A	*6	#8
2019	19.GP02.B	*7	#9
2019	19.GP02.C	*6	#8
2019	19.GP02.D	*7	#9
2019	19.GP02.E	*6	#8
2019	19.GP02.F	*7	#9
2019	19.GP02.G	*7	#9
2019	19.GP02.H	*7	#9
2019	19.GP02.I	*7	#9
2019	19.GP02.J	*7	#9
2019	19.GP02.K	*7	#9
2019	19.GP02.L	*6	#8
2019	19.GP02.M	*7	#9
2019	19.GP02.N	*7	#9
2019	19.SD02.A	*8	#10
2019	19.SD02.B	*8	#10
2019	19.SD02.C	*5	#10
2019	19.SD02.D	*8	#10
2019	19.SD02.E	*8	#10
2019	19.PB41.A	*9	#8
2019	19.SD14.A	*1	#11
2019	19.SD14.B	*10	#12
2019	19.PB45.A	*11	#13
2019	19.SH01.A	*10	#7
2019	19.SH01.B	*12	#14
2019	19.SH01.C	*12	#7
2019	19.SD03.A	*13	#14
2019	19.SD03.B	*13	#15
2019	19.SD03.C	*13	#15
2019	19.SD04.A	*14	#15
2019	19.SD04.B	*15	#14
2019	19.SD04.C	*14	#7
2019	19.SD04.D	*14	#15
2019	19.SD04.E	*14	#15
2019	19.SD27.A	*16	#4
2019	19.SD05.A	*17	#13
2019	19.SD05.B	*18	#16
2019	19.SD05.C	*18	#16
2019	19.SD05.D	*18	#16
2019	19.SD05.E	*18	#16
2019	19.SD05.F	*17	#13
2019	19.SD05.G	*17	#13
2019	19.SD05.H	*17	#13
2019	19.PB52.A	*1	#5
2019	19.PB69.A	*18	#17
2022	22.SH01.A	*1	#1
2022	22.SH01.B	*1	#1
2022	22.SH01.C	*1	#1
2022	22.SH01.D	*1	#1
2022	22.SH01.E	*1	#1
2022	22.SH01.F	*1	#1
2022	22.SH01.G	*1	#1
2022	22.SH01.H	*1	#1
2022	22.SH02.A	*2	#2
2022	22.SH02.B	*3	#2
2022	22.SH02.C	*3	#2
2022	22.SH02.D	*2	#2
2022	22.SH02.E	*3	#2
2022	22.SH02.F	*2	#2
2022	22.SH02.G	*2	#2
2022	22.SH03.A	*4	#3
2022	22.SH03.B	*4	#3
2022	22.SH03.C	*4	#3
2022	22.SH03.D	*4	#3
2022	22.SH03.E	*4	#3
2022	22.SH03.F	*4	#3
2022	22.SH03.G	*4	#3
2022	22.SH04.A	*5	#4
2022	22.SH04.B	*5	#4
2022	22.SH04.C	*5	#4
2022	22.SH04.D	*5	#4
2022	22.SH04.E	*5	#4
2022	22.SH05.A	*6	#5
2022	22.SH05.B	*6	#5
2022	22.SH05.C	*6	#5
2022	22.SH05.D	*6	#5
2022	22.SH05.E	*6	#5
2022	22.SH05.F	*6	#5
